# Performance analysis of an indoor visible light communication system using LED configurations and diverse photodetectors

**DOI:** 10.1038/s41598-025-99643-z

**Published:** 2025-05-08

**Authors:** Fayza M. Elamrawy, Ahmed Abd El Aziz, Salah Khamis, Hossam Kasem

**Affiliations:** 1 Department of Basic Science, Pharos University, Alexandria, Egypt; 2 Electronics and Communication Engineering Department, College of Engineering and Technology, Arab Academy for Science, Technology and Maritime Transport, Alexandria, Egypt; 3 Electronics and Electrical communications, Faculty of Engineering, Tanta University, Tanta, Egypt; 4 Computer science and Engineering, Faculty of Engineering, Egypt-Japan University of science and Technology (E- JUST), New borg El-Arab, Alexandria, Egypt

**Keywords:** VLC, Arduino, LED array, BPW34 photodiode, Solar cell, LDR, Electrical and electronic engineering, Lasers, LEDs and light sources

## Abstract

This study evaluates the implementation and performance of a Visible Light Communication (VLC) system designed to transmit text data between two personal computers (PCs). The proposed system utilizes three distinct light-emitting diode (LED) —Single LED, 2 × 2 LED Array, and 4 × 4 LED Array—as transmitters and three types of photodetectors—BPW34 photodiode, solar cell, and light-dependent resistor (LDR)—as receivers. Controlled by an Arduino, the system investigates the relationships between data rate, transmission distance, beam angle, LED configurations, and detector types. *Experimental results confirmed that the BPW34 photodiode outperformed the solar cell and LDR in terms of maximum achievable data rate and transmission distance*,* consistent with its superior bandwidth and sensitivity characteristics*. The findings also reveal that, at a zero-degree beam angle, the system employing the BPW34 photodiode achieved a maximum transmission distance of 9.15 m at 1 kilobit per second (kbps) with a single LED configuration, diminishing to 1.24 m at 4 kbps. The 2 × 2 LED array extended the distance 14.20 m at 1 kbps and 2.30 m at 4 kbps. Conversely, the 4 × 4 LED array exhibited the most extensive propagation distances, achieving 16.30 m at 1 kbps and maintaining 3.25 m at 4 kbps. These findings underscore the efficacy of increasing LED array size in enhancing propagation distance, particularly at lower data rates. The study highlights the potential of VLC systems for efficient indoor optical communication, providing valuable insights for optimizing LED configurations and receiver technologies in future applications.

## Introduction

Visible Light Communication (VLC) is an emerging optical wireless communication technology that uses visible light to transmit data. As an alternative to conventional radio frequency (RF) communication systems, VLC operates in the visible light spectrum (380–750 nm)^1^, offering significant advantages such as higher data rates, enhanced security, and reduced interference^2^. VLC systems can utilize existing lighting infrastructure, making them both energy-efficient and cost-effective, while their inherent line-of-sight propagation offers enhanced security by reducing the risk of eavesdropping^3^. VLC is distinct from other optical communication technologies, such as infrared (IR) communication, due to its ability to handle high-speed data transfer and dual functionality, where the same device can provide both illumination and communication^4^.

The last decade has witnessed a significant progress in VLC applications^5^, including its integration with healthcare systems, the development of VLC-based Internet of Things (IoT) systems, and its exploration for autonomous vehicles and vehicle-to-infrastructure communication. VLC has become a mature technology that is expected to play a critical role in next-generation wireless communication systems,, particularly as part of 5G^6^and upcoming 6G systems^7^.

In healthcare, VLC has proven useful for secure communication due to its immunity to electromagnetic interference (EMI), making it particularly advantageous in hospitals where RF-based communication could interfere with medical devices such as pacemakers or MRI machines^8^. VLC is also being explored for use in body sensor networks (BSNs) to provide real-time patient monitoring and secure data transmission^9^. Additionally, VLC plays a crucial role in the development of smart home systems and the broader IoT ecosystem^10^. As smart devices proliferate, VLC offers an efficient and interference-free communication medium for IoT devices that require high-speed, low-latency connectivity. VLC can also be integrated into smart lighting systems to support communication between devices in home automation systems^11^.

VLC has become a critical technology for vehicular communication systems, such as vehicle-to-vehicle (V2 V)^12^and vehicle-to-infrastructure (V2I) networks^13^. These systems rely on VLC for real-time communication between vehicles and traffic infrastructure, enhancing road safety and supporting autonomous driving features. VLC offers a reliable, low-latency communication medium that is less susceptible to interference compared to RF systems.

VLC is increasingly integrated into the broader 5G and 6G communication frameworks, where it is used to complement traditional RF-based systems. In 5G, VLC helps alleviate congestion in the RF spectrum and provides high-capacity backhaul solutions^14^. In 6G, VLC is expected to enable ultra-high-speed communication, with applications in wireless data centers, high-definition video transmission, and high-density IoT networks^15^. VLC’s line-of-sight requirement and high signal security make it an ideal solution for public safety applications, including secure communications in law enforcement, military, and government sectors. VLC networks can operate without interference from other RF systems, ensuring secure and reliable communication in critical situations^16^.

The rapid growth of VLC systems is driven by advancements in LEDs and photodetectors, enabling high-speed optical communication. VLC utilizes LEDs as transmitters, rapidly switching between on and off states to encode data while maintaining their primary illumination function. Silicon PIN photodiodes, such as the BPW34, serve as efficient receivers, converting modulated light signals into electrical signals.

White LEDs are preferred for VLC due to their availability, energy efficiency, and dual function in lighting and communication^17^. They typically consist of a blue LED chip with a yellow phosphor coating^18^, producing broad-spectrum visible light (380–750 nm) compatible with standard photodetectors. With high luminous efficacy (100–150 lm/W) and low power consumption, they are widely used in indoor VLC, vehicular communication, and smart lighting applications. Additionally, LED arrays enhance transmission power and range by increasing optical intensity^19^, improving signal reception, and mitigating environmental interference.

The BPW34 silicon PIN photodiode is well-suited for VLCdue to its high sensitivity, fast response, low cost^20^, and broad spectral range (430–1100 nm)^21^. Despite its peak sensitivity at 950 nm, it effectively detects white LED signals. Its 7.5 mm² photosensitive area and ± 65° sensitivity angle support both line-of-sight and non-line-of-sight communication^22^. With low dark current (2 nA at 10 V) and microsecond-scale response time, it enables high-speed modulation schemes such as On-Off Keying (OOK), ensuring accurate optical-to-electrical conversion even under varying light intensities.

Despite the growing interest in VLC as a cost-effective and energy-efficient solution for indoor optical communication, challenges remain in optimizing system performance across varying transmission distances, data rates, and receiver types. Existing studies often focus on single configurations or exhibit limitations in providing a comparative analysis of detector types and LED array designs. This work aims to bridge this gap by investigating the performance of a VLC system using different LED configurations (Single LED, 2 × 2 LED Array, and 4 × 4 LED Array) and receivers (BPW34 photodiode, solar cell, and LDR). By analyzing the relationships between data rate, transmission distance, and beam angle, this study seeks to provide practical insights for optimizing LED configurations and receiver technologies, ultimately enhancing the reliability and efficiency of VLC systems for indoor applications.

## Methodology

The methodology section outlines the design, configurations, and program flow of the proposed VLC system under investigation.

### Experimental setup

The experimental setup aims to achieve two primary objectives: coverage analysis and performance optimization. For coverage analysis, the study evaluates the field of view (FOV) by varying transmission distances and angles, identifying the system’s maximum boundary under different transmitter and receiver configurations. Performance optimization focuses on examining how factors such as LED array size and receiver type influence system performance, while exploring trade-offs between transmission distance, data rate, coverage angle, and signal reliability to identify the optimal configurations for specific applications.

#### System design

**Fig. 1:**
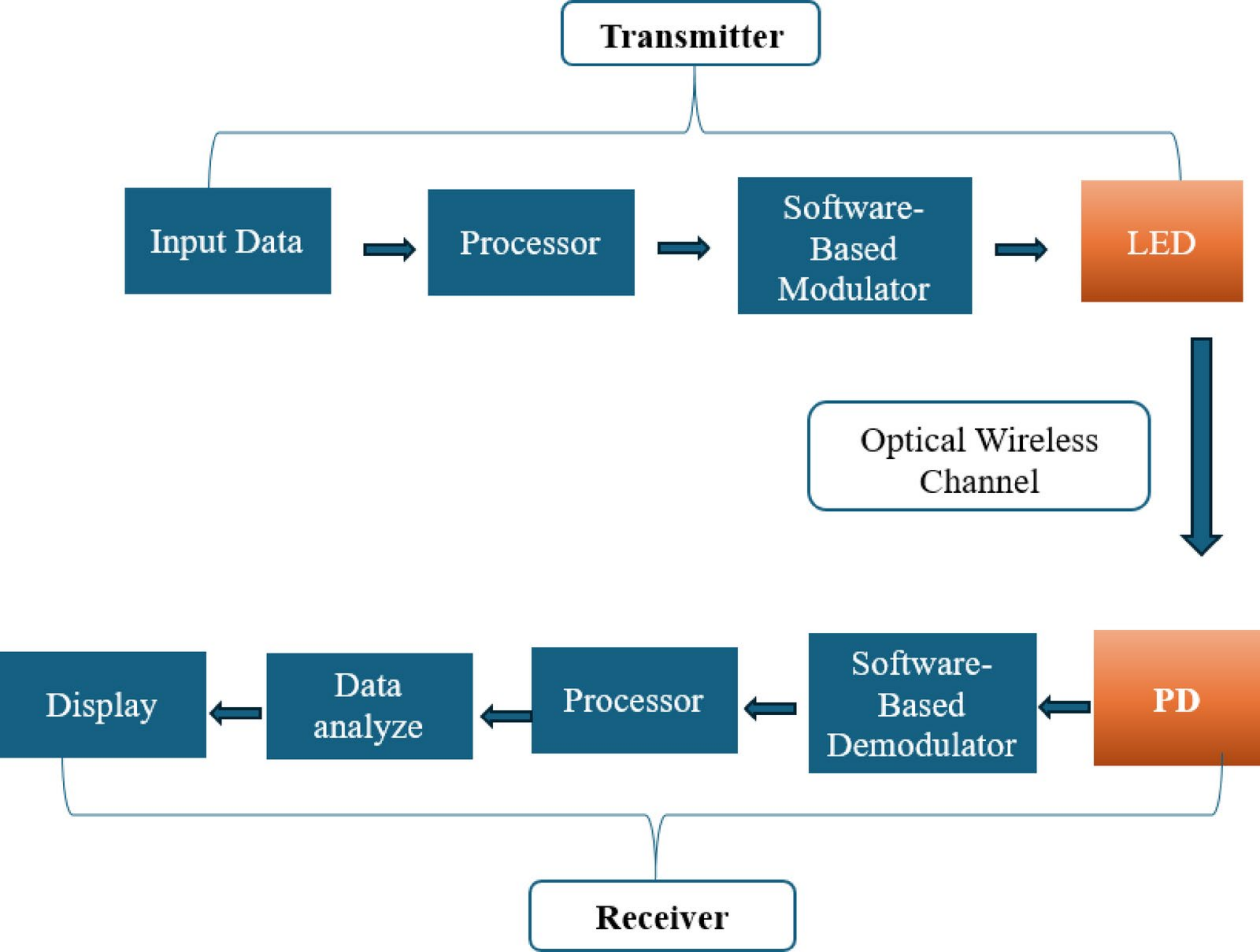
System block diagram representing the modular design for data processing, transmission, and reception.

Figure 1 represents the block diagram of the proposed VLC system, which employs a modular architecture for data processing, transmission, and reception to facilitate the investigation of different design parameters. The process begins with raw input data, which is encoded and prepared for transmission using an Arduino Uno microcontroller. The encoded data is passed through a modulator employing On-Off Keying (OOK), a binary modulation technique where light presence (1) and absence (0) encode data. The modulated signal is then emitted by the LED-based transmitter, propagating through free space to the receiver. At the receiving end, the transmitted light signals are captured by a photodetector. The photodetector converts the light into electrical signals, which are subsequently processed to remove noise and decode the original data. This modular design allows for flexibility and iterative experimentation with various LED arrays and photodetector configurations to optimize system performance.

#### Transmitter configurations

**Fig. 2:**
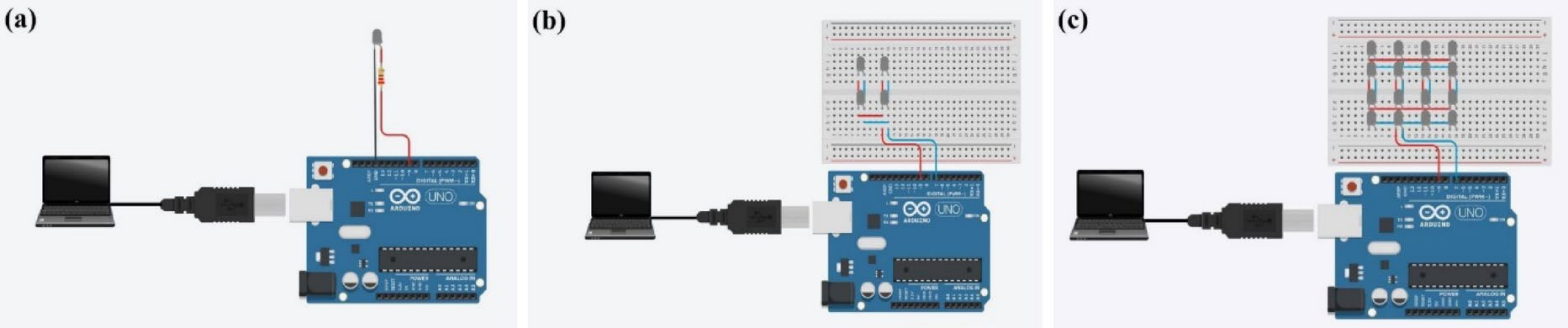
Transmitter configurations with (**a**) single LED, (**b**) 2 × 2 LED array, and (**c**) 4 × 4 LED array.

The transmitter setup utilizes white LEDs as the primary light source. To ensure sufficient light intensity and extended coverage, the study employs three distinct configurations of LEDs: a single LED, a 2 × 2 LED array, and a 4 × 4 LED array. These configurations are visually represented in Fig. 2. The baseline configuration features a single LED (Fig. 2a), which offers limited light output and coverage. Due to the relatively low luminance of a single LED, arrays were introduced to improve system performance. Larger arrays not only enhance light intensity but also provide redundancy and adaptability under dynamic conditions. For this reason, the 2 × 2 LED array (Fig. 2b) is employed to enhance both light intensity and coverage by combining four LEDs into a compact arrangement. Additionally, the 4 × 4 LED array (Fig. 2c) is arranged in a square grid comprising 16 LEDs to maximize light output and significantly broaden the coverage area. These configurations are tested to evaluate their impact on transmission distance, data rate, coverage angle, and signal reliability.

The transmitter’s primary role is to modulate and emit light signals based on the encoded data. The Arduino Uno microcontroller interfaces with the LED driver circuit to control the modulation and ensure precise signal generation. The use of OOK modulation simplifies the encoding process, making it efficient for binary data transmission. This modulation technique is chosen for its simplicity and reliability in converting electrical signals into optical pulses.

#### Receiver configurations

**Fig. 3:**
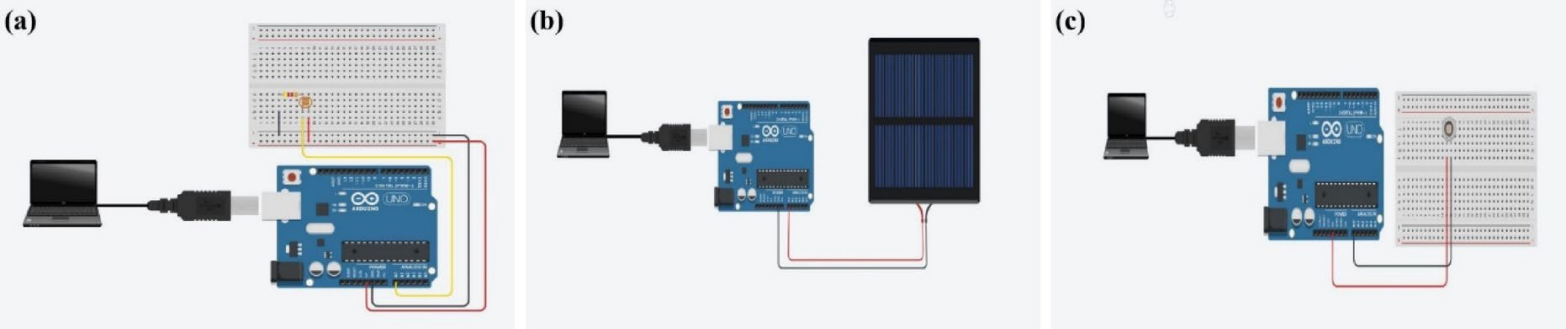
Receiver configurations with (**a**) LDR, (**b**) Solar Cell, and (**c**) BPW34 Photodiode.

The receiver’s role is to detect and decode light-based signals reliably. The receiver setup employs three distinct types of receivers—Light-Dependent Resistor (LDR), solar cell, and BPW34 photodiode—each selected to comprehensively evaluate their performance and potential applications in VLC systems under different light conditions. The receiver configurations are represented in Fig. 3.

The LDR (Fig. 3a) is a passive sensor that alters its electrical resistance in response to the intensity of incident light. Made primarily from photoconductive materials like cadmium sulfide (CdS), its resistance decreases as the light intensity increases. This behavior makes LDRs well-suited for detecting gradual changes in light levels, such as in ambient light sensors. However, their relatively slow response time limits their use in high-speed data transmission. In the context of VLC, the LDR is tested for its ability to detect light modulated signals. While it performs adequately in low-speed systems, its lag in response time renders it less effective for high-data-rate communication.

The solar cell (Fig. 3b) serves as the second type of receiver in the experimental setup. As a photovoltaic device, the solar cell converts incident light into electrical energy through the photovoltaic effect. Typically made from silicon, solar cells are highly sensitive to light intensity and can generate significant electrical output under strong illumination. Their large surface area allows them to capture substantial amounts of light, making them ideal for systems transmitting high-power light signals. However, the solar cell’s response time is moderate and less suitable for high-speed data communication, as it struggles with the rapid modulation required for transmitting binary data in VLC systems. Despite this, its versatility and sensitivity make it a valuable component for specific use cases involving high-intensity light.

The most advanced receiver used in the experiment is the BPW34 photodiode (Fig. 3c), a high-speed silicon-based device designed for detecting light signals with high precision. This photodiode operates by converting light into an electrical current, with its output directly proportional to the intensity of the incident light. The BPW34 is particularly suited for VLC applications due to its exceptional speed and precision. With a peak sensitivity in the near-infrared spectrum and strong performance in visible light ranges, it seamlessly integrates with white LED transmitters. Unlike LDRs and solar cells, the BPW34’s fast response time allows it to accurately decode high-frequency modulated signals, making it the ideal receiver for high-speed VLC systems.

Each of these receivers offers distinct strengths and limitations. The LDR is inexpensive and effective for low-speed applications but lacks the speed required for advanced systems. The solar cell’s ability to handle high light intensities is offset by its slower response time. In contrast, the BPW34 photodiode provides the necessary speed and precision for modern VLC systems, making it the most suitable choice for applications demanding high data rates and efficient performance. This comparative analysis highlights the diverse capabilities of these receivers and their roles in optimizing VLC performance across different scenarios.

Each receiver is connected to the Arduino to captures and decode the light pulses into electrical signals, subsequently processed to reconstruct the original data. The three receiver configurations are analyzed for sensitivity, response time, noise resistance, and suitability under varying lighting conditions. The modular nature of the system allowed for direct comparisons, enabling the selection of the optimal receiver for specific applications.

Beam angle measurements are conducted exclusively using the BPW34 photodiode, as it exhibits sufficient sensitivity for angular displacement experiments. To maintain consistency, the center of the LED source is initially aligned with the center of the BPW34 photodiode in a straight-line configuration. The LED source remains fixed throughout the experiments, while the photodiode is incrementally positioned at different angles relative to the source. This setup allows for a systematic analysis of how the reception angle influences the maximum propagation distance.

### Program flow and data transmission

#### Transmitter operation

The transmitter program, implemented on an Arduino Uno, is designed to encode textual data into binary format and modulate the LED using OOK modulation. The operation begins with input data preparation, where textual information is converted into its ASCII representation. Each ASCII character is further transformed into an 8-bit binary format using modular arithmetic, with the resulting binary bits stored for controlling the LED’s state.

For modulation, the binary data is transmitted as light pulses using OOK. In this scheme, a binary “1” corresponds to the LED being turned on (HIGH), while a binary “0” corresponds to the LED being turned off (LOW). Each bit is transmitted with a predefined delay in microseconds to ensure signal clarity. The Arduino manages this process by controlling the LED connected to a certain pin, referred to as LEDPIN. After completing the transmission of the binary-encoded data, the LED is turned off to signify the end of the signal.

This modular approach allows the Arduino Uno to handle data preparation, binary encoding, and light signal modulation effectively, enabling reliable transmission of the encoded data as modulated light pulses. This functionality forms the core of the VLC system’s transmitter operation.

#### Receiver operation

The receiver setup in the VLC system is responsible for capturing and decoding the transmitted light pulses, converting them into electrical signals for accurate data reconstruction. The process begins with signal detection, where a photodetector (such as LDR, solar cell, or BPW34 photodiode) continuously monitors the incoming light signals. The detected signals are subjected to threshold-based decoding, with those exceeding a predefined threshold value interpreted as binary “1”, while lower signals are identified as binary “0”. To ensure proper decoding, the receiver program synchronizes with the start bit of the data sequence. Once synchronized, the binary sequence is converted back into its ASCII equivalent, effectively reconstructing the original transmitted data. The decoded information is then displayed on the Serial Monitor, allowing for analysis and verification of the communication system’s performance.

### Statistical analysis of data rate and transmission distance

To ensure the reliability of the measured data rate and transmission distance, each experimental setup was tested 10 times per measurement point under identical conditions. The standard deviation (SD) was computed to quantify measurement variability, and due to variations across different conditions, it is reported as a percentage of the mean value rather than in absolute units.

For data rate (R), the SD was computed as:

$$\:{SD}_{R}=\sqrt{\frac{1}{N}\sum\:_{i=1}^{N}{\left({R}_{i}-\stackrel{-}{R}\right)}^{2}}$$

where N is the number of trials, R_i_​ is the measured data rate in each trial, and $\:\stackrel{-}{R}$ is the mean data rate.

Similarly, for transmission distance (d), the SD was determined using:

$$\:{SD}_{d}=\sqrt{\frac{1}{N}\sum\:_{i=1}^{N}{\left({d}_{i}-\stackrel{-}{d}\right)}^{2}}$$

Where d_i​_ represents the measured transmission distance in each trial, and $\:\stackrel{-}{d}$ is the mean transmission distance.

Since the variability differs across measurement conditions, the percentage standard deviation (%SD) is used for better representation and is calculated as:

$$\:\%{SD}_{R}=\left(\frac{{SD}_{R}}{\stackrel{-}{R}}\right)\times\:100$$

$$\:\%{SD}_{d}=\left(\frac{{SD}_{d}}{\stackrel{-}{d}}\right)\times\:100$$

Given the small magnitude of the percentage SD relative to the graph scales, numerical values are reported in the figure captions instead of being displayed as error bars.

## Results and discussion

**Fig. 4:**
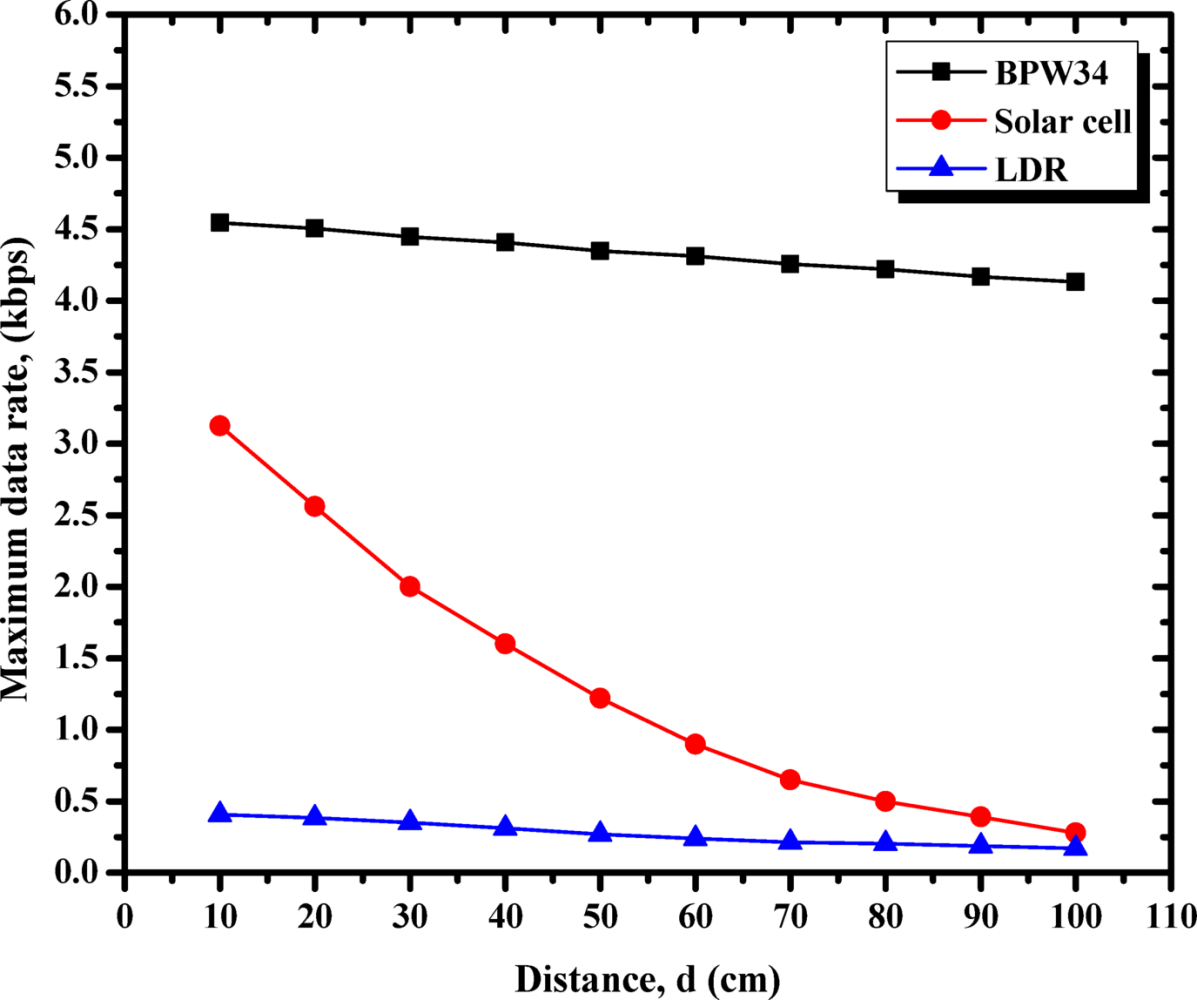
Maximum data rate vs. transmission distance for different photodetectors (BPW34 photodiode, solar cell, and LDR). [The %SD_R_ in data rate ranges from 0.7–3.2%].

The study initiates with the presentation of Fig. 4, which systematically evaluates the three proposed photodetectors to determine the optimal receiver. This assessment focuses on the maximum achievable data rates devoid of errors, thereby facilitating the selection of the most appropriate photodetector for further analysis and exploration throughout the research. Figure 4 demonstrates the performance of three proposed photodetectors—BPW34 photodiode, solar cell, and LDR—across varying transmission distances, ranging from 10 cm to 100 cm. Among these, the BPW34 photodiode consistently achieves the highest maximum data rate, maintaining superior performance even at longer distances. The solar cell, while starting with a moderate data rate, shows a sharp decline as the distance increases, highlighting its sensitivity to transmission range. In contrast, the LDR exhibits the lowest data rates across all distances with minimal variation, indicating its limitations for high-speed communication. These results underscore the photodiode’s reliability and robustness, especially for longer-distance VLC applications, while the solar cell and LDR are better suited for short-range, low-speed scenarios.

**Fig. 5:**
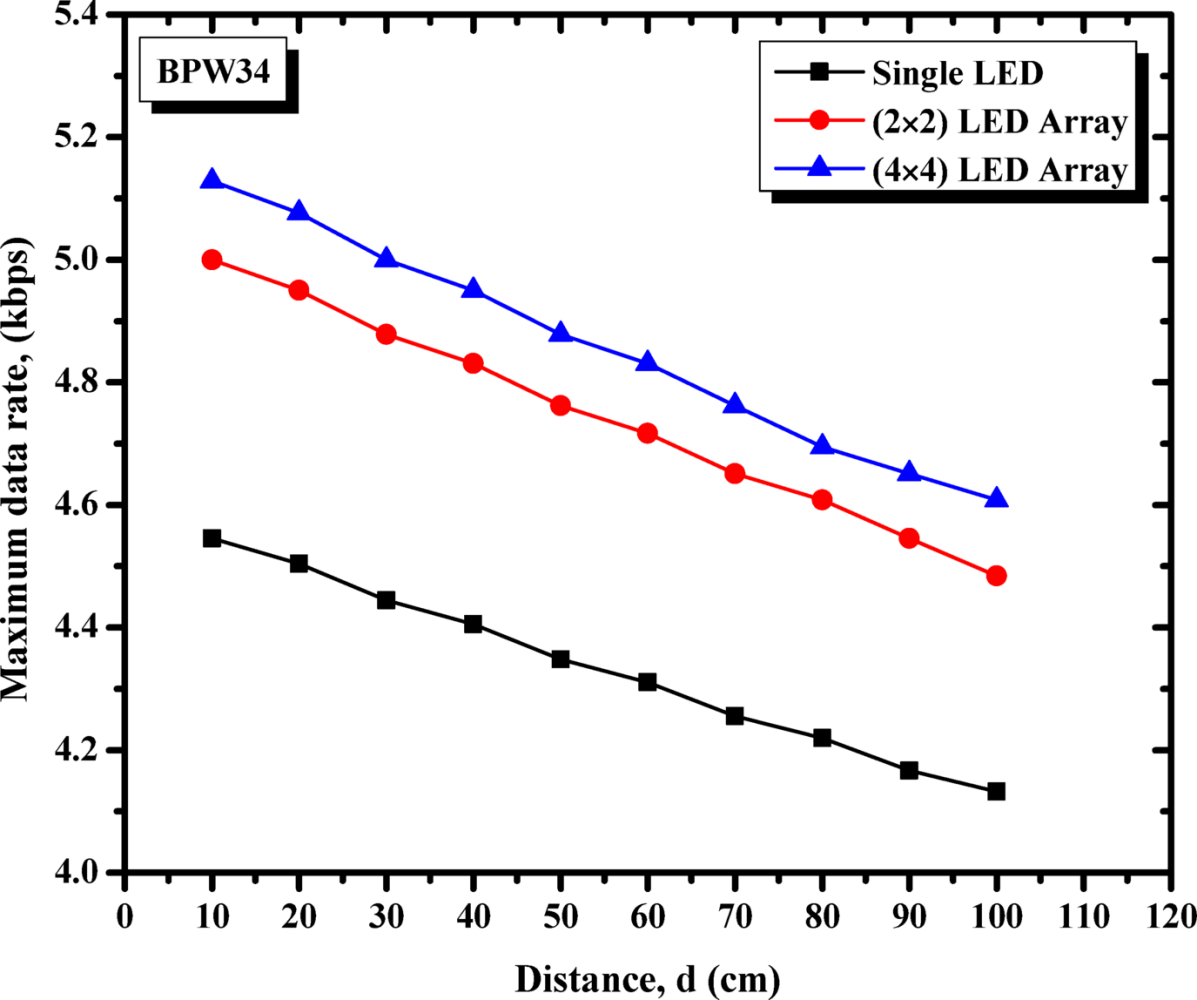
Maximum data rate vs. transmission distance for different LED configurations (single LED, 2 × 2 LED array, and 4 × 4 LED array). [The %SD_R_ in data rate ranges from 0.7–3.2%].

After identifying the BPW34 photodiode as the most suitable photodetector, the study proceeded to investigate the effect of LED array size on system performance. Figure 5 illustrates this comparison by evaluating the maximum data rate of three LED configurations—single LED, 2 × 2 LED array, and 4 × 4 LED array—across varying transmission distances. The 4 × 4 LED array consistently outperforms the other configurations, achieving the highest data rates across all distances. The 2 × 2 LED array ranks second, while the single LED configuration exhibits the lowest data rates. The results reveal that larger LED arrays benefit from higher light intensity and better utilization of the communication channel, which improves signal reliability and extends communication range. The rate of decline in data rate with distance is similar across all configurations; however, larger arrays maintain higher absolute values, particularly at distances greater than 50 cm. This indicates the effectiveness of larger arrays in maintaining performance over extended ranges.

**Fig. 6:**
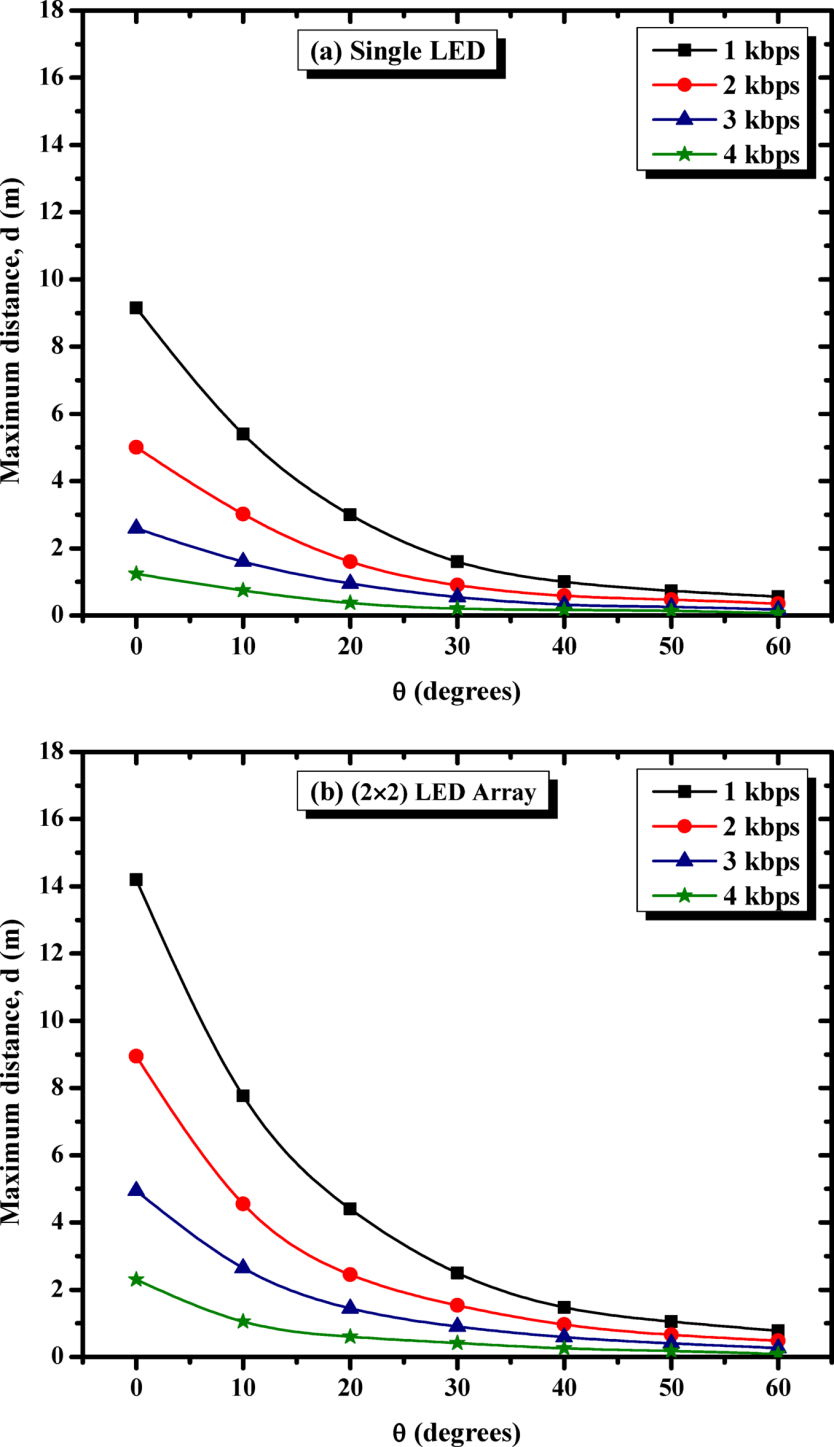

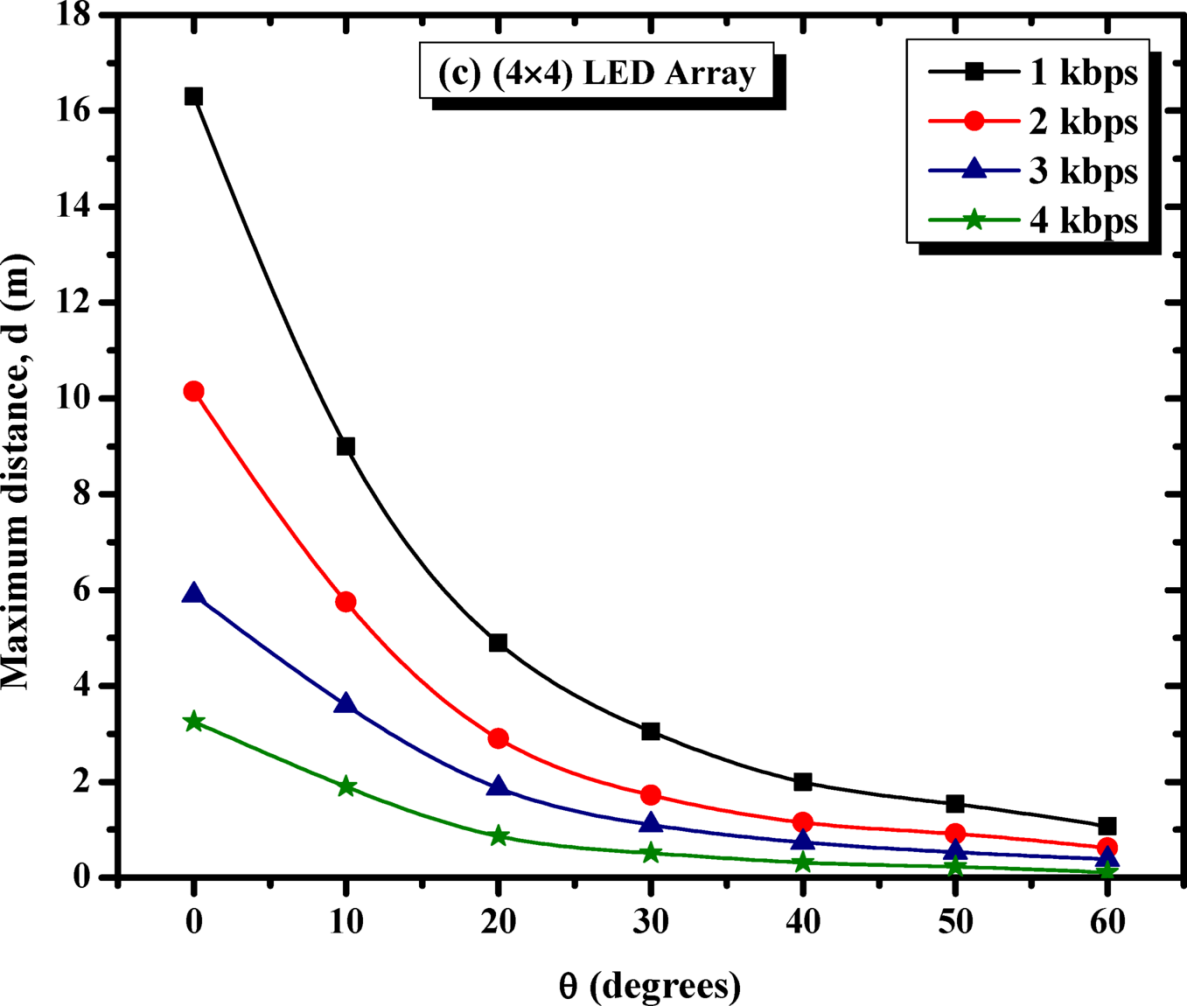
Effect of beam angle and data rate on maximum propagation distance for different LED configurations (**a**) single LED, (**b**) 2 × 2 LED array, and (**c**) 4 × 4 LED array. [The %SD_d_ in transmission distance ranges from 1.3–4.5%].

The study subsequently focused on examining the impact of beam angle and data rate on system performance. Figure 6 examines how these factors influence the maximum propagation distance for three LED configurations: (a) single LED, (b) 2 × 2 LED array, and (c) 4 × 4 LED array. The analysis includes data rates of 1 kbps, 2 kbps, 3 kbps, and 4 kbps, providing insights into the trade-offs between beam angle, data rate, and propagation range for each configuration.

As can be seen in Fig. 6a, the single LED configuration shows a noticeable decline in propagation distance as the beam angle widens. At a beam angle of 0°, the system achieves its longest propagation distances, with 1 kbps reaching about 9.15 m. However, as the data rate increases to 4 kbps, the maximum distance reduces significantly to 1.24 m due to increased signal attenuation. At wider angles (e.g., 30° and 60°), the distances drop further for all data rates, with the decline being more pronounced at higher rates. This configuration demonstrates the limitations of single LEDs for achieving extended coverage in VLC systems, particularly at higher data rates and wider beam angles.

The 2 × 2 LED array outperforms the single LED configuration by providing greater propagation distances across all beam angles and data rates, as shown in Fig. 6b. At 0°, the system achieves 14.2 m at 1 kbps, with distances gradually decreasing to around 2.30 m at 4 kbps. The beam angle significantly affects performance, with a noticeable decline in distances as the angle widens to 60°. Compared to the single LED, the 2 × 2 array maintains better performance at higher data rates and wider angles, attributed to the increased light intensity provided by the array. This configuration balances extended coverage and reasonable performance degradation at wider angles.

On the other hand, Fig. 6c demonstrates that the 4 × 4 LED array exhibits the best performance among the three configurations, achieving the longest propagation distances at all data rates. At 0°, the array reaches over 16.30 m at 1 kbps, outperforming the other configurations. Even at 4 kbps, the system maintains a distance of 3.25 m. However, like the other configurations, the propagation distance decreases as the beam angle widens. At 60°, the array still achieves significant distances compared to the single LED and 2 × 2 array, demonstrating its superior light output and resilience to angular variation. This makes the 4 × 4 LED array the most effective configuration for maintaining communication over extended distances and wide beam angles.

Figure 6 highlights key trends influencing VLC performance. As the beam angle widens from 0° to 60°, the propagation distance decreases across all configurations and data rates due to reduced light intensity reaching the receiver. Lower data rates, such as 1 kbps, consistently achieve longer distances, whereas higher data rates, such as 4 kbps, experience significant attenuation, limiting their effective communication range. Among the configurations, larger LED arrays, particularly the 4 × 4 array, outperform smaller setups by maintaining greater distances across all angles and data rates, attributed to their higher light output and broader coverage. These findings emphasize the need to optimize LED array size, beam angle, and data rate to achieve effective VLC communication, with the 4 × 4 LED array proving to be the most suitable choice for applications requiring extended distances and wider beam angles.

**Fig. 7:**
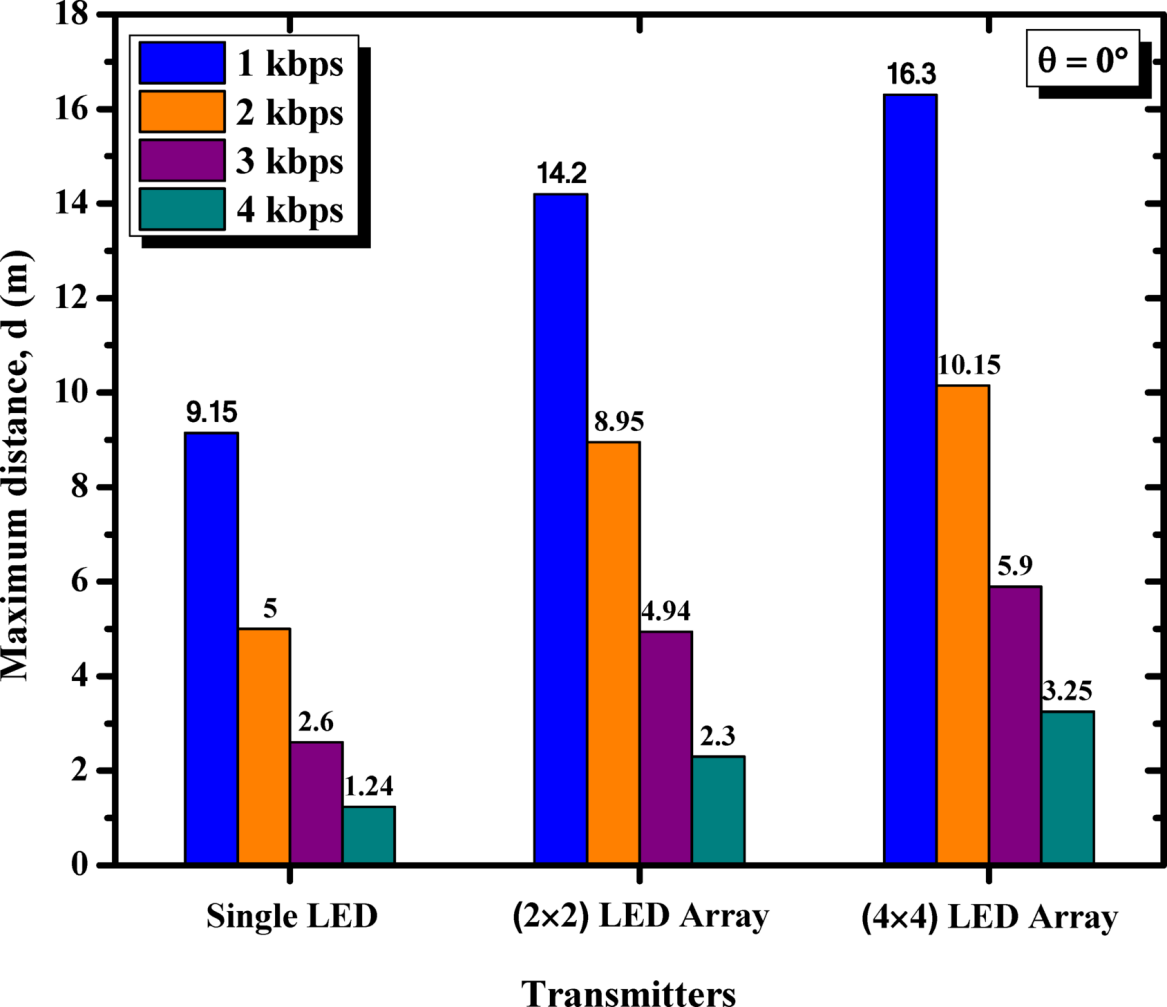
Maximum propagation distances at different data rates for all LED configurations (single LED, 2 × 2 LED array, and 4 × 4 LED array). [The %SD_d_ in transmission distance ranges from 1.3–4.5%].

Figure 7 presents a histogram comparing the maximum propagation distances achieved by single LED, 2 × 2 LED array, and 4 × 4 LED array configurations across various data rates ranging from 1 kbps to 4 kbps. The results confirm the superior performance of the 4 × 4 LED array, which consistently achieves the longest distances at all data rates, followed by the 2 × 2 LED array. In contrast, the single LED configuration demonstrates limited performance, particularly at higher data rates. This comparison underscores the substantial advantages of utilizing larger LED arrays, especially for maintaining reliable communication at lower data rates. The inclusion of this histogram provides a clear visual representation of the relationship between LED array size, data rate, and propagation distance, offering valuable clarification and support for the observed trends in system performance.

**Fig. 8:**
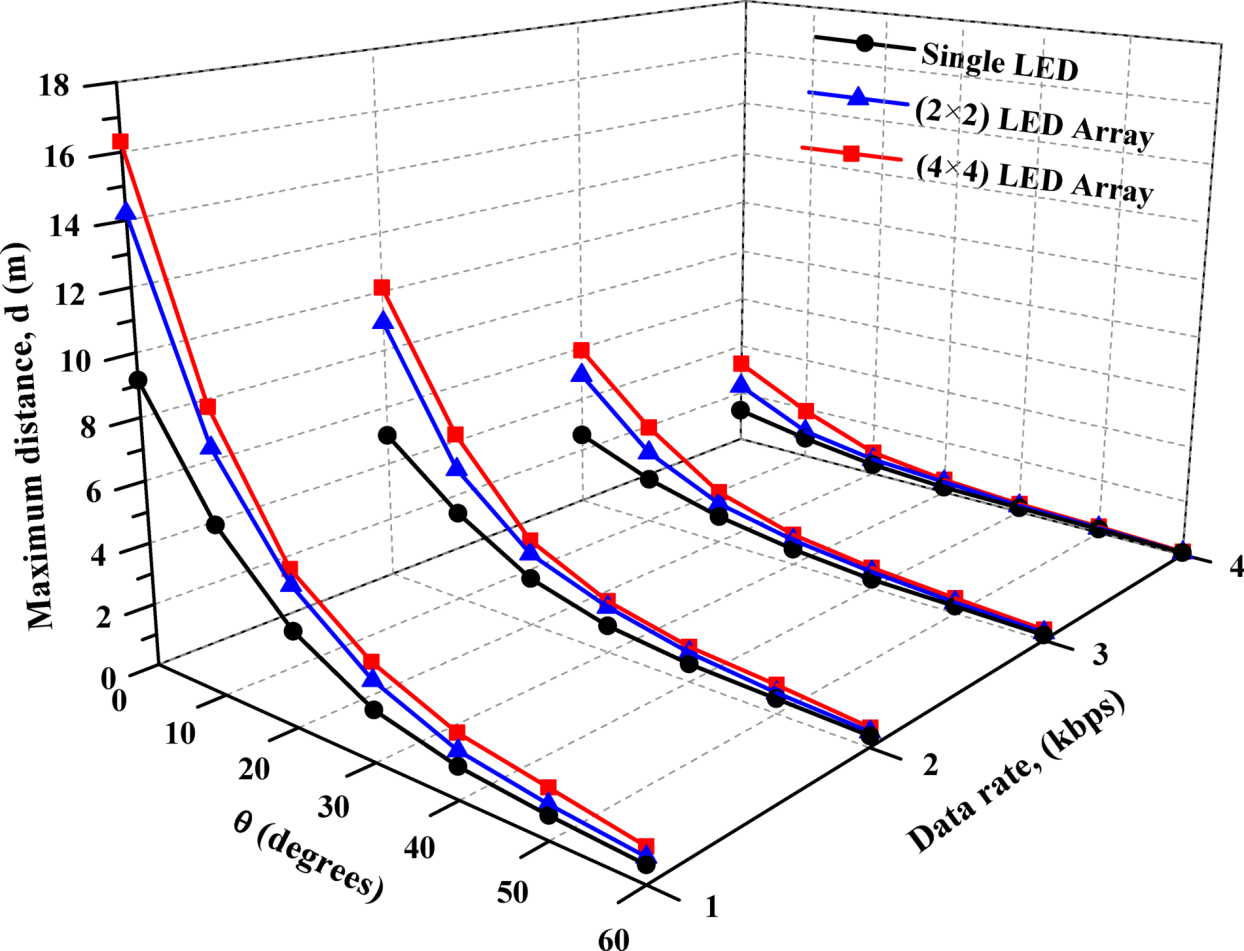
Analysis of maximum distances and beam angles for different LED configurations and data rates. [The %SD_d_ in transmission distance ranges from 1.3–4.5%].

Figure 8 provides a comprehensive analysis of the maximum propagation distances achieved at various beam angles for different LED configurations and data rates, integrating all the critical parameters into a single graph. This unified representation offers a clear understanding of the interdependence between all parameters, allowing for a complete evaluation of system performance. The graph demonstrates that larger LED arrays, such as the 4 × 4 configuration, consistently outperform smaller configurations by maintaining greater propagation distances across all beam angles and data rates. It also highlights how wider beam angles and higher data rates significantly reduce the maximum achievable distances, underscoring the trade-offs between coverage area and communication speed. Including all these variables in one graph allows for an effective comparison, facilitating a deeper understanding of the optimal conditions for maximizing VLC system performance. This approach aids in drawing robust conclusions about the advantages of larger LED arrays and the need to balance beam angle and data rate to achieve reliable communication over extended distances.

## Conclusion

This study evaluated the performance of a VLC system for transmitting text data between two personal computers, utilizing LEDs as transmitters and BPW34 photodiode, solar cell, and LDR as receivers. The findings demonstrate that the BPW34 photodiode is the most effective receiver, achieving superior detection capabilities with maximum distances reaching 16.30 m at lower data rates and maintaining connectivity at a signal reception angle of up to 60 degrees. Among the LED configurations, the 4 × 4 LED array consistently outperformed the single LED and 2 × 2 LED array by delivering the longest propagation distances and most robust signal reliability, particularly at lower data rates.

The study further reveals a clear trade-off between beam angle and propagation distance, with wider angles significantly reducing maximum transmission range across all configurations. These results emphasize the importance of optimizing LED array size and beam angle for enhancing VLC performance in indoor applications, where line-of-sight communication and efficient use of existing lighting infrastructure are critical.

The proposed VLC system showcases the potential for reliable and efficient indoor optical communication, offering a cost-effective and secure alternative to traditional wireless technologies. Future research could focus on further optimizing LED configurations and improving photodetector sensitivity to overcome challenges associated with higher data rates and to expand the applicability of VLC systems in advanced indoor environments, including smart homes, IoT networks, and healthcare facilities.

## Data Availability

All data generated or analyzed during this study are included in this published article.
